# Prediction of BP Reactivity to Talking Using Hybrid Soft Computing Approaches

**DOI:** 10.1155/2014/762501

**Published:** 2014-09-21

**Authors:** Gurmanik Kaur, Ajat Shatru Arora, Vijender Kumar Jain

**Affiliations:** Electrical and Instrumentation Engineering Department, Sant Longowal Institute of Engineering & Technology, Deemed University (Established by Government of India), Longowal, Sangrur District, Punjab 148106, India

## Abstract

High blood pressure (BP) is associated with an increased risk of cardiovascular diseases. Therefore, optimal precision in measurement of BP is appropriate in clinical and research studies. In this work, anthropometric characteristics including age, height, weight, body mass index (BMI), and arm circumference (AC) were used as independent predictor variables for the prediction of BP reactivity to talking. Principal component analysis (PCA) was fused with artificial neural network (ANN), adaptive neurofuzzy inference system (ANFIS), and least square-support vector machine (LS-SVM) model to remove the multicollinearity effect among anthropometric predictor variables. The statistical tests in terms of coefficient of determination (*R* ^2^), root mean square error (RMSE), and mean absolute percentage error (MAPE) revealed that PCA based LS-SVM (PCA-LS-SVM) model produced a more efficient prediction of BP reactivity as compared to other models. This assessment presents the importance and advantages posed by PCA fused prediction models for prediction of biological variables.

## 1. Introduction

Accurate measurement of BP is essential in epidemiological studies, in screening programmes, in research studies, and in clinical practice to classify individuals, to ascertain hypertension related risks (coronary heart disease, stroke, and kidney failure), and to guide management. Recommendations of several international organisations including the American Heart Association (AHA) [1], British Hypertension Society (BHS) [2], and European Society of Hypertension (ESH) [3] revealed that accuracy of BP measurements is highly associated with the conditions in which the measurements are taken. The observer should be aware of the considerable variability that may occur in BP due to various factors. However, it is not always feasible to control all the factors, but we can minimize their effect by taking them into account in reaching a decision [3].

In clinical practice, talking is one of the most common measurement disturbances influencing BP measurement accuracy [4]. It can contribute to elevated BP reading, termed BP reactivity to talking, that may result in the misdiagnosis of hypertension or in overestimation of the severity of hypertension and may lead to overly aggressive therapy. Antihypertensive treatment may be unnecessary in the absence of concurrent cardiovascular risk factors [5].

In the past few years, several studies have quantified the effect of talking on BP. Zheng et al. [6] measured BP in healthy subjects under five different conditions including resting, deeper breathing, talking, and head and arm movement and proved that SBP and DBP changed significantly in comparison to the resting condition. Le Pailleur et al. [7] explored a sharp and significant increase in SBP and DBP of hypertensive subjects while talking. Le Pailleur et al. [8] showed an instantaneous rise in SBP and DBP of treated and untreated hypertensive patients under a period of stress talking and a period of counting aloud (active periods).

Zheng et al. [9] demonstrated significantly higher manual and automated MAPs with talking in healthy subjects. Lynch et al. [4] reported that verbal activity is consistently associated with marked elevations in both normotensive and hypertensive subjects. Tardy et al. [10] demonstrated that talking state increased the BP as compared to resting state of the subjects. Lynch et al. [11] described sudden extreme drop in blood pressure in both experimental and clinical situations when a person is talking about or describing situations of hopelessness and helplessness. Long et al. [12] showed statistically significant increase in BP when speaking compared to when quiet. Hellmann and Grimm [13] investigated the effect of talking on subjects with one previous diastolic blood pressure reading of 90 mm Hg or more and not taking antihypertensive medicines. Blood pressure increased significantly under both talking conditions (reading neutral material for part of the procedure and reading neutral material continuously).

Epidemiological studies from different populations have explored a significant correlation between BP and anthropometric characteristics [14–16]. Therefore, anthropometric variables should be considered to attain an accurate measurement of BP. However, multicollinearity between anthropometric predictor variables has also been reported, which may result in “overfitting” of the prediction model [17–19]. One approach to dealing with multicollinearity is to use PCA, a statistical approach. By using PCA the original data set can be transformed into principal components (PCs) that are orthogonal and are able to explain the maximal variance of the data without losing any information [20, 21].

Soft computing covers computational techniques that offer somewhat “inexact” solutions of very complex problems through modeling and analysis with a tolerance of imprecision, uncertainty, partial truth, and approximation. The successful applications of soft computing approaches in biomedical studies suggest that the impact of soft computing will be felt increasingly in the coming years.

The fusion of a statistical and soft computing approach usually improves the training speed, enhances the robustness of the model, and reduces the calibration error. These models may aid the clinicians in the decision-making process regarding clinical admission, early prevention, early clinical diagnosis, and application of clinical therapies. In this sense, this paper focuses on the development of PCA based soft computing approaches for prediction of BP reactivity to talking, which include conventional statistical method of PCA for data preprocessing. We developed PCA based ANN (PCA-ANN), PCA based ANFIS (PCA-ANFIS), and PCA-LS-SVM models for prediction of BP reactivity to talking in normotensive and hypertensive subjects. The prediction accuracy of developed models was assessed and compared using statistical indices including coefficient of determination (*R* ^2^), root mean square error (RMSE), and mean absolute percentage error (MAPE) to select the model that most accurately predicts the BP reactivity.

The rest of the paper is structured as follows. In Section 2, we present the details of data collection.  Section 3 deals with the experimental approaches used for data analysis.  Section 4 deals with the summary of results obtained.  Section 5 describes the discussion and Section 6 concludes with future directions of work.

## 2. Data Collection

A total of 40 normotensive and 30 hypertensive female subjects among the students, staff, and faculty of Sant Longowal Institute of Engineering and Technology (Deemed University), Longowal, District Sangrur, Punjab, India, volunteered for this study. Eligible participants had to be over 18 years of age. We excluded the subjects who were pregnant and who had arrhythmias. The institutional research committee approved the research protocol and all participants gave written informed consent before participation.

A standard questionnaire was administrated to collect information on anthropometric characteristics of the participants. A specially separated room was used to conduct the study. This ensured minimal interference within the room while the tests were being carried out. The observers involved in the study were trained using the BHS's BP measurement training materials [22].

To eliminate observer bias, BP was measured using a clinically validated (under standardized measurement conditions), newly purchased, and fully automated sphygmomanometer OMRON HEM-7203 (OMRON HEALTHCARE Co., Ltd., Kyoto, JAPAN) that uses the oscillometric method of measurement. The BP monitor is available with a small cuff (17–22 cm), medium cuff (22–32 cm), and large cuff (32–42 cm). The appropriate size of cuff was determined from the mid-arm circumference of the subject.

Subjects were advised to avoid alcohol, cigarette smoking, coffee/tea intake, and exercise for at least 30 minutes prior to their BP measurement. They were instructed to empty their bladder and sit upright with elbows on table, supported back, and feet flat on the ground, as they are the potential confounding factors. Moreover, they were asked not to talk and move during measurement [1].

After a rest period of 5 minutes [1], the measurements were performed four times repeatedly at an interval of one minute. First measurement was discarded and the average of last three measurements was taken into account. Subsequently, the same measurement protocol was repeated under talking phase during which the observer asked each subject to “tell me about your work in detail” [4]. During talking phase, the observer only talked to the subject to maintain the flow of conversation, making every possible effort to talk minimally. To improve the reliability of measurements, the subjects were examined for a week [3].

## 3. Experimental Methods

Data were expressed as mean ± SD. A paired *t*-test was used to assess the difference between measurements of resting and talking conditions.

### 3.1. PCA

Firstly, Bartlett's test of sphericity [23] and Kaiser Meyer Olkin (KMO) measure of sampling adequacy [24] were applied to check the suitability of data for application of PCA.

Bartlett's test of sphericity tests the null hypothesis that the correlation matrix is an identity matrix or there is no relationship between predictor variables. Consider

(1) χ2=−{[N−1]−[2K+56]}log⁡e|R|,

where χ2 is chi-square, *N* is sample size, *K* is number of predictor variables, log⁡_*e*_ is natural log, and |*R*| is determinant of the correlation matrix.

KMO compares the magnitude of calculated correlation coefficients and partial correlation coefficients. The formula for KMO is given as follows:

(2) $$\begin{array}{c} \text{KMO}=\frac{\sum_{i\neq j}^{}\sum r_{ij}^{2}}{\sum_{i\neq j}^{}\sum r_{ij}^{2}+\sum_{i\neq j}^{}\sum a_{ij}^{2}}, \end{array}$$

where ∑_*i*≠*j*_ is sum over all variables in the matrix when variable *i* ≠ *j*, *r*_*ij*_ is Pearson correlation coefficient between variables *i* and *j*, and *a*_*ij*_ is partial correlation coefficient between variables *i* and *j*.

KMO index ranges, from 0 to 1, should be greater than 0.6 for the PCA to be considered appropriate.

PCA is a multivariable statistical analysis technique. The objective of PCA is to remove the multicollinearity problem and reduce the number of predictor variables and transform them into PCs which are independent linear combinations of the original data set and account for the maximum possible variance of the original data set so that adequate information from the original data set can be extracted [20, 21].

The eigenvalues of the standardized matrix are calculated from

(3) $$\begin{array}{c} \left|C-\lambda I\right|=0, \end{array}$$

where *C* is correlation matrix of the standardized data, *λ* is eigenvalues, and *I* is identity matrix. The weights of the variables in the PCs are then obtained by

(4) $$\begin{array}{c} \left|C-\lambda I\right|W=0, \end{array}$$

where *W* is matrix of weights.

To evaluate the influence of each predictor variable in the PCs, varimax rotation was used to obtain values of rotated factor loadings. These loadings represent the contribution of each predictor variable in a specific PC. The PCs used for the prediction of BP reactivity to unsupported back were obtained through multiplication of the standardized data matrix by weights (*W*) [25].

### 3.2. ANN

ANN's customary architecture was composed of an input layer, an output layer, and one or more intervening layers, also referred to as hidden layers to capture the nonlinearity in data.

**Figure 1:**
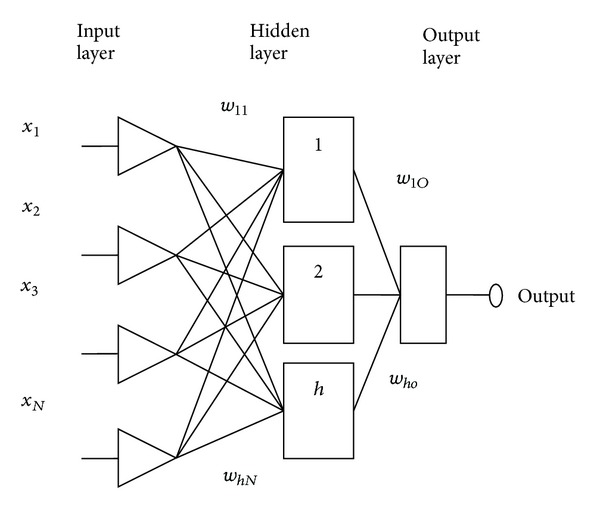
Architecture of ANN.

Figure 1 shows an ANN model consisting of *N* nodes in input layer, one hidden layer with *h* hidden nodes, and an output layer with one node.

Network is trained by presenting one pair of input-output vector at a time. The weighted sum of inputs calculated at *t*th hidden node is

(5) $$\begin{array}{c} {\text{NET}}_{t}=\overset{N}{\underset{i=1}{\sum }}w_{ti}x_{i}+b_{t}, \end{array}$$

where *w*_*ti*_ is weight on connection from the *i*th to the *t*th node, *x*_*i*_ is input data from input node, *N* is total number of input nodes, and *b*_*t*_ is bias on the *t*th hidden node.

Each hidden node uses a tangent sigmoid transfer function to generate an output, say *Z*_*t*_, between 0 and 1. The outputs from each hidden nodes, along with the bias *b*_0_ on the output node, send to the output node and weighted sum becomes

(6) $$\begin{array}{c} \text{NET}=\overset{h}{\underset{t=1}{\sum }}v_{t}Z_{t}+b_{0}, \end{array}$$

where *h* is total number of hidden nodes and *v*_*t*_ is weight from the *t*th hidden node to the output node.

The weighted sum NET becomes the input to the linear transfer function of the output node and the predicted output is

(7) $$\begin{array}{c} \hat{Y}=f\left(\text{NET}\right). \end{array}$$

And then the second phase of the BP algorithm, adjustment of the connection weights, begins. The parameters of the ANN can be determined by minimizing the following objective function in the training process:

(8) $$\begin{array}{c} \text{SSE}=\overset{n}{\underset{j=1}{\sum }}{\left(y_{i}-{\hat{Y}}_{j}\right)}^{2}, \end{array}$$

where ${\hat{Y}}_{j}$ is output of the network from *j*th observation.

The sensitivity *S*_*i*_ of the outputs to each of the *i*th inputs, as partial derivatives of the output with respect to the input, under the assumption that relationship of *Y* and *X* is monotone [26], is given as

(9) Si=  ∂Y^∂Xi=∑t=1h∂Y^∂NET∂NET∂Zt∂Zt∂NETt∂NETt∂Xi=∑t=1h[f′(NET)vtf′(NETt)wti]

or

(10) $$\begin{array}{c} {\hat{S}}_{i}=\overset{h}{\underset{t=1}{\sum }}v_{t}w_{ti} \end{array}$$

with the assumption that *f*′(NET) and *f*′(NET)_*t*_ are constants. The independent variable with higher relative positive or negative sensitivity has the higher positive or negative impact on dependent variable.

### 3.3. ANFIS

**Figure 2:**
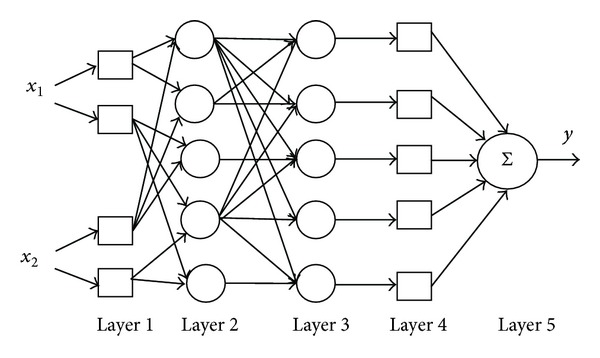
Architecture of ANFIS.

ANFIS, a multilayer feed forward network, uses neural network learning algorithms and fuzzy reasoning to map an input space to an output space, as shown in Figure 2. It has the ability to combine the verbal power of a fuzzy system with the numeric power of a neural network.

It can construct an input-output mapping based on human knowledge (if-then fuzzy rules) and stipulated input-output data pairs. The parameters of membership function, if-then rule exertion, and output parameters are calculated by training data set. The training algorithm is usually hybrid or back propagation. The ANFIS implements the rules of the form

  *R*_*r*_ : If *x*_1_ is *A*_*j*1_^(1)^ … and *x*_*n*_ is *A*_*jn*_^(*n*)^, then
  (11) $$\begin{array}{c} y=\alpha_{0}^{\left(r\right)}+\alpha_{1}^{\left(r\right)}\cdot x_{1}+\cdots +\alpha_{n}^{\left(r\right)}\cdot x_{n}, \end{array}$$

where *x*_1_ is independent variable, *A*_*j*1_^(1)^ is a fuzzy linguistic concept, and *y* is dependent variable.

*Input Layer (L*_1_ *).* Each unit of input layer stores parameters of membership functions to define a membership function that represents a linguistic term. 

*Input Membership Function Layer (L*_2_ *).* Each unit of this layer represents a rule. The inputs to a unit *R*_*r*_ ∈ *L*_2_ are degrees of membership which are multiplied to determine the degree of fulfilment *τ*_*r*_ for the rule represented by *R*_*r*_. 

*Logical Nodes (L*_3_ *).* This layer consists of a unit for each rule *R*_*r*_ that computes relative degree of fulfilment as follows:

(12) $$\begin{array}{c} {\overset{-}{\tau }}_{r}=\frac{\tau_{r}}{\sum_{R_{i}\in L_{2}}^{}\tau_{i}}. \end{array}$$

Each unit of *L*_3_ is connected to all the rule units in *L*_2_. 

*Output Membership Function Layer (L*_4_ *).* Each unit of *L*_4_ computes the output of a rule *R*_*r*_ as

(13) $$\begin{array}{c} O_{r}={\overset{-}{\tau }}_{r}\left(\alpha_{0}^{\left(r\right)}+\alpha_{1}^{\left(r\right)}\cdot x_{1}+\cdots +\alpha_{n}^{\left(r\right)}\cdot x_{n}\right). \end{array}$$

The units *L*_4_ are connected to all units of input layer and to exactly one unit in *L*_3_. 

*Output Layer (L*_5_ *).* It computes the final output *y* by adding all the outputs from *L*_4_ [27].

### 3.4. LS-SVM

**Figure 3:**
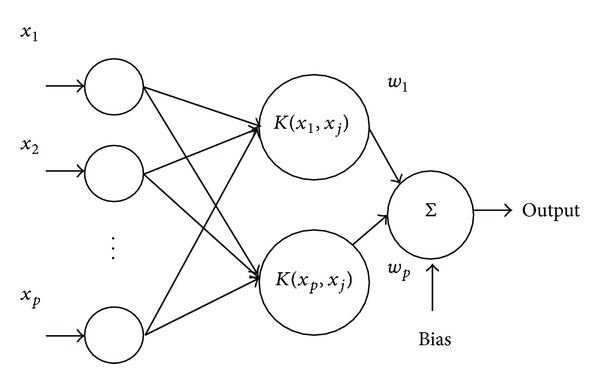
Architecture of LS-SVM.

LS-SVM is an extension of standard support vector machine. It converts the inequality constraints of SVM into equality ones which leads to solving a linear system instead of a quadratic problem, whose convergence speed is faster [28]. It has been widely used in estimation and approximation of function [29]. The architecture of LS-SVM is shown in Figure 3.

Given a set of training data set

(14) $$\begin{array}{c} \left(x_{i\;},y_{i}\right),\;i=1,2,\ldots ,l,\;\;x_{i}\in R^{2},\;\;y_{i}\in R \end{array}$$

with the input vector *x*_*i*_ and the output vector *y*_*i*_, the regression function of least square-support vector machine, in feature space *F*, can be stated as

(15) $$\begin{array}{c} y\left(x\right)=w^{T}\emptyset \left(x\right)+b, \end{array}$$

where *w* is weight vector and *b* is bias. *∅*(*x*) maps the input *x* into a vector in *F*.

The model is inferred from the training data set by minimizing the cost function given below

(16) $$\begin{array}{c} \frac{1}{2}w^{T}w+\frac{\gamma }{2}\overset{l}{\underset{i=1}{\sum }}e_{i}^{2}, \end{array}$$

subject to equality constraint

(17) $$\begin{array}{c} y_{i}=w^{T}\emptyset \left(x_{i}\right)+b+e_{i}, \end{array}$$

where *e*_*i*_ is error, *i* = 1, 2, 3,…, *l*, and  *γ* is regularization parameter.

Solving this optimization problem in dual space leads to finding the coefficients *α*_*i*_ and *b* in the following solution:

(18) $$\begin{array}{c} y\left(x\right)=\overset{l}{\underset{i=1}{\sum }}\alpha_{i}K\left(x_{i},x\right)+b, \end{array}$$

where *K*(*x*_*i*_, *x*) is kernel function.

### 3.5. Performance Indices Used for Model Comparison

For the comparison of developed models and selection of the optimal among them, performance of models was evaluated using *R* ^2^ and RMSE and MAPE.

*R* ^2^ is the square of the correlation coefficient between two variables *x* and *y* whose *n* pairs are available as follows:

(19) $$\begin{array}{c} R^{2}=1-\frac{\sum_{i=1}^{n}{\left(y_{i}-y_{di}\right)}^{2}}{\sum_{i=1}^{n}{\left(y_{di}-y_{m}\right)}^{2}}. \end{array}$$

RMSE is the square root of mean square error, given by following equation:

(20) $$\begin{array}{c} \text{RMSE}=\sqrt{\frac{1}{n}\overset{n}{\underset{i=1}{\sum }}{\left(y_{i}-y_{di}\right)}^{2}}. \end{array}$$

MAPE is defined as average of percentage errors, given by following equation:

(21) $$\begin{array}{c} \text{MAPE}=\frac{100}{n}\overset{n}{\underset{i=1}{\sum }}\left|\frac{y_{i}-y_{di}}{y_{i}}\right|, \end{array}$$

where *n* is the number of samples, *y*_*i*_ is the predicted value obtained from the model, *y*_*di*_ is the actual value, and *y*_*m*_ is the average of the actual values.

The lower the RMSE and MAPE, the better the accuracy of the model in predicting the parameter. Also, the highest *R* ^2^ values indicated that the model performed the best [30].

## 4. Results

Descriptive statistics for each anthropometric characteristic is given as mean and SD in Table 1.

The results of paired *t*-test demonstrated statistically significant higher SBP, DBP, and mean arterial pressure (MAP), (*P* < 0.001) in talking condition. The mean rise was found to be higher in hypertensive individuals than normotensives, as shown in Table 2. These results are consistent with the recommendations of AHA for BP measurement in humans and experimental animals [1].

Table 3 presents the Pearson's correlation coefficients calculated for all anthropometric variables. High values of correlation coefficient (greater than 0.6) between pairs of anthropometric characteristics [31] revealed the existence of multicollinearity.

Before applying PCA, Bartlett's test of sphericity and Kaiser-Meyer-Olkin (KMO) measure of sampling adequacy were applied to determine whether PCA was suitable for data studied. The results are shown in Table 4. High value of chi-square (χ2) for Bartlett's test suggests that use of PCA is appropriate (*P* < 0.001) in normotensive and hypertensive subjects. The value of KMO is also greater than 0.6 which indicates that our sample size is enough to apply PCA [32].

The first four PCs (PC1–PC4), explaining more than 5% of total variation, as shown in Table 5, were retained for further analysis.

Rotated component loadings after varimax rotation represent the extent to which the original anthropometric characteristics are influential in forming PCs, as shown in Table 6.

The bold marked loads show the highest correlation between anthropometric characteristic and corresponding component. For both normotensive and hypertensive subjects, weight and BMI were positively highly correlated with PC1 and a negative high correlation between height and PC2 was observed.

Principal score values for assigned PCs were determined by using principal score coefficients.

Moreover, the value of Pearson's correlation (correlation coefficient < 0.6) between PCs, as shown in Table 7, indicates the elimination of multicollinearity effect presented in Table 3.

To develop PCA based soft computing prediction models 80% of data were used for training while entire data set was used for testing. Moreover, data must be normalized to achieve more accurate predictions [38]. The predicted BP reactivity values were denormalized for comparison with the actual values. MATLAB 7.5 version was used to develop the prediction models.

### 4.1. PCA-ANN

To achieve the best ANN structure for BP reactivity prediction, various structures of feed-forward neural network with different number of neurons in hidden layer were investigated. Finally, with consideration of statistical indices, a structure with two hidden layers, having six nodes in each hidden layer, was developed. There were four input nodes representing the four PCs and one output node representing the BP reactivity to talking. Tangent sigmoid and linear transfer functions were used as activation functions in the hidden and output layers. Back propagation learning algorithm based on Levenberg-Marquardt technique was used [39].

**Figure 4:**
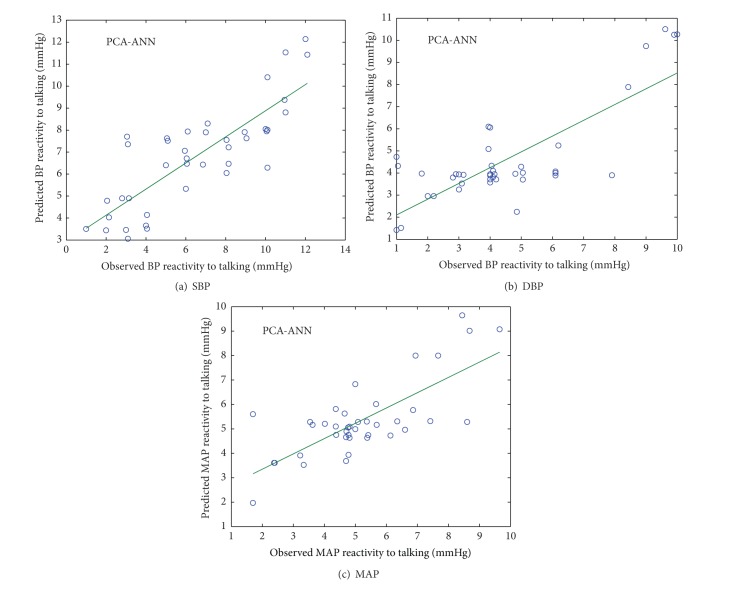
Scatter plot between observed and predicted values of SBP, DBP, and MAP reactivity of normotensive subjects using PCA-ANN model.

**Figure 5:**
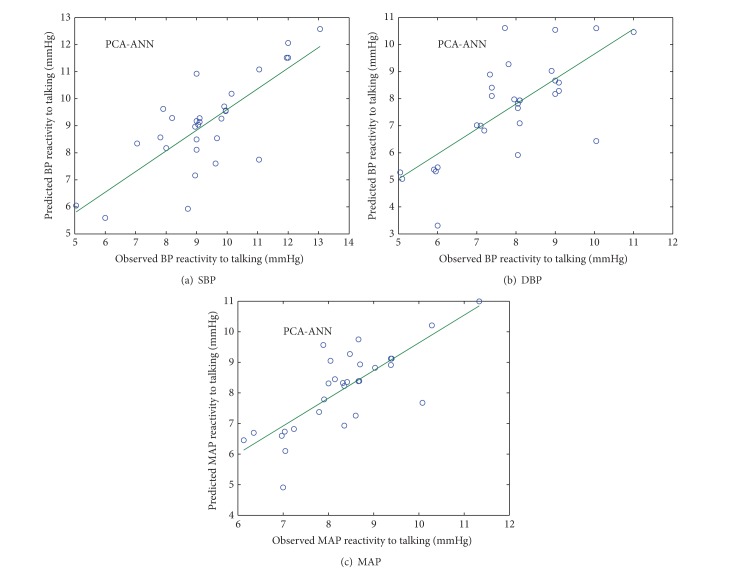
Scatter plot between observed and predicted values of SBP, DBP, and MAP reactivity of hypertensive subjects using PCA-ANN model.

Figures 4 and 5 show the scatter plot between observed and predicted values of SBP, DBP, and MAP reactivity from PCA-ANN model in normotensive and hypertensive subjects, respectively.

### 4.2. PCA-ANFIS

PCA-ANFIS model was developed using genfis1 with grid partition on data. Different ANFIS parameters were tested in order to achieve the perfect training and maximum prediction accuracy.

Input membership functions “trapmf” and “gauss2mf” were used to predict SBP and DBP reactivities, respectively, in normotensive individuals, whereas membership function “psigmf” was used to predict SBP and DBP reactivity in hypertensive individuals. Output membership function “linear” was used.

Other parameters of trained PCA-ANFIS model were number of membership functions = 16, number of nodes = 55, number of linear parameters = 80, number of nonlinear parameters = 32, total number of parameters = 112, and number of fuzzy rules = 16.

**Figure 6:**
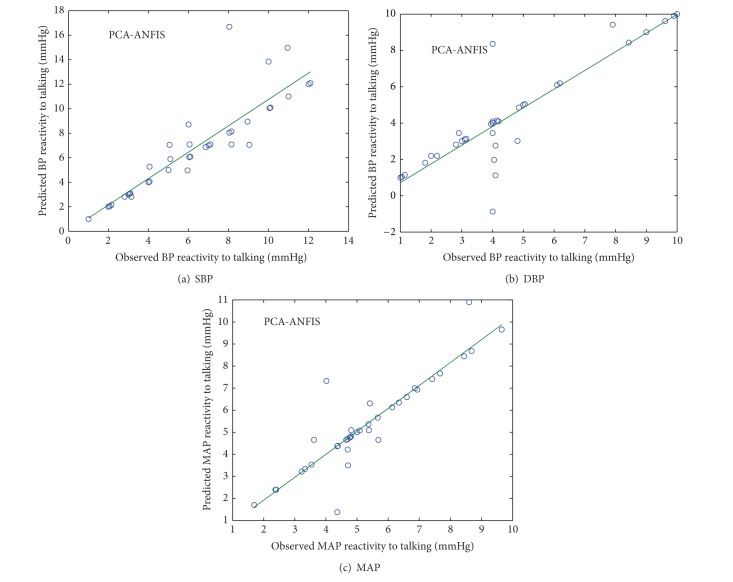
Scatter plot between observed and predicted values of SBP, DBP, and MAP reactivity of normotensive subjects using PCA-ANFIS model.

**Figure 7:**
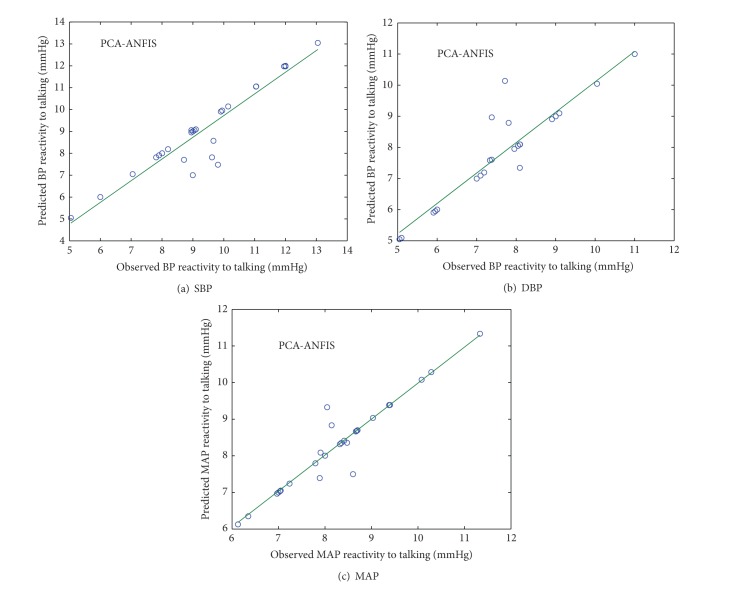
Scatter plot between observed and predicted values of SBP, DBP, and MAP reactivity of hypertensive subjects using PCA-ANFIS model.

The observed and predicted values of SBP, DBP, and MAP reactivity from PCA-ANFIS model for normotensive and hypertensive subjects were plotted in Figures 6 and 7.

### 4.3. PCA-LS-SVM

A PCA-LS-SVM model using RBF kernel and grid search optimization algorithm with 2-fold cross-validation was developed to obtain the optimal parameter combination [40]. The optimal values of *γ* (regularization parameter) and *σ* ^2^ (squared bandwidth) for normotensive and hypertensive subjects were shown in Table 8.

**Figure 8:**
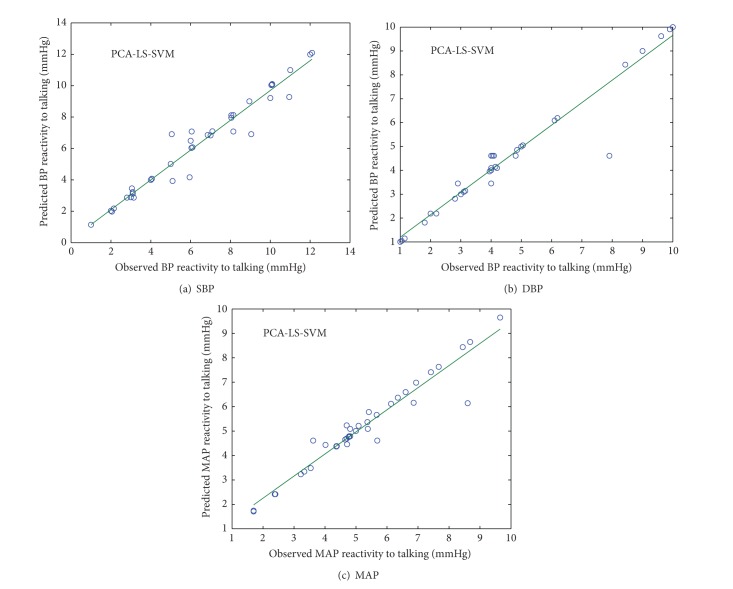
Scatter plot between observed and predicted values of SBP, DBP, and MAP reactivity of normotensive subjects using PCA-LS-SVM model.

**Figure 9:**
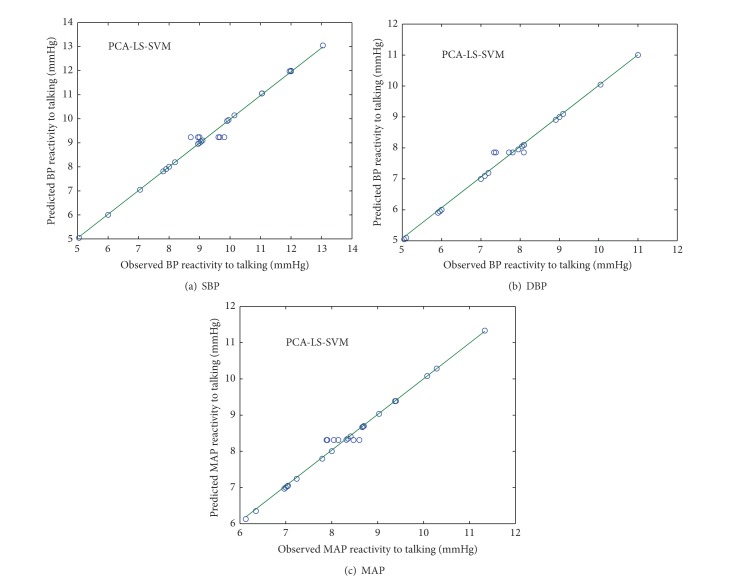
Scatter plot between observed and predicted values of SBP, DBP, and MAP reactivity of hypertensive subjects using PCA-LS-SVM model.

Figures 8 and 9 show the scatter plot between observed and predicted values of SBP, DBP, and MAP reactivity from PCA-LS-SVM model in normotensive and hypertensive subjects, respectively.

Comparison of statistical indices for the models, as shown in Table 9, revealed that PCA-LS-SVM model has the highest value of R2 and lowest value of RMSE for the prediction of BP reactivity to talking in normotensive and hypertensive subjects.

## 5. Discussion

For proper diagnosis and treatment of hypertension, accurate and reproducible BP measurements are essential.

This study confirms and extends previous studies [4, 6–13] by documenting a significant increase in BP with talking. This finding tends to support Weiner et al. [41] suggestion that there may be an association between verbal activity and BP elevations. And withdrawal from such verbal activity has important clinical implications for the cardiovascular system.

Furthermore, we illustrated an application of PCA based soft computing models in predicting the BP reactivity to talking. PCA corrects for confounding caused by anthropometric characteristics including age, height, weight, BMI, and AC and, therefore, normotensive subjects were used to provide a basis for comparison.

As far as we know, this paper is the first study related to prediction of BP reactivity to talking using PCA based soft computing approaches. Therefore, the results were compared with indirectly related studies [33–37], as shown in Table 10. Promising results of soft computing techniques in all studies are due to their high degree of robustness and fault tolerance. In this work, specifically, the best performance of LS-SVM is sourced from its several advantages including global optimal solution ability, fast convergence rate, and good generalization with small size sample.

This study has a number of advantages. We used small, medium, and large size cuffs, which may have produced more accurate readings. And we took the mean of multiple readings to strengthen the accuracy of BP measurements.

However, any single comparison between the models might not reliably represent the true results. Validation of the computing models using larger database is essential to get an accurate measure of performance outside the development population.

## 6. Conclusion

The successful development of any prediction model depends largely on the quality and nature of data used for model development. To address the issue of multicollinearity within the anthropometric variables, PCA is incorporated. Furthermore, performance comparison of PCA-ANN, PCA-ANFIS, and PCA-LS-SVM models revealed the potential capability of PCA-LS-SVM model in predicting BP reactivity. This work may provide a valuable reference for researchers and engineers who apply soft computing models for modeling biological variables. The results are helpful in physician's diagnosis for the prevention of hypertension in clinical medicine. Our future research is targeted to study an ensemble approach by combining the outputs of different hybrid techniques with more predictor variables and larger data sets to achieve wide clinical application of the soft computing.

## Figures and Tables

**Table 1:** Descriptive characteristics of anthropometric characteristics of study sample.

Anthropometric characteristic	Normotensive		Hypertensive	
	Mean	SD	Mean	SD
Age (year)	23.1	1.24	42.83	6.665
Height (m)	1.61	0.03	1.583	0.035
Weight (kg)	55.96	7.29	62.48	10.89
BMI (kg/m^2^)	21.55	2.504	23.57	3.497
AC (cm)	26.56	2.45	26.72	2.4

**Table 2:** Results of paired *t*-test.

Subjects	BP, mm Hg	Mean difference ± SD	*t*	*P*	95% CI of mean difference
Normotensives	SBP	6.31 ± 2.409	16.567	<0.001	5.540 to 7.081
	DBP	5.857 ± 1.584	23.388	<0.001	5.350 to 6.363
	MAP	6.008 ± 1.231	30.854	<0.001	5.164 to 6.402

Hypertensives	SBP	9.634 ± 1.283	41.117	<0.001	9.154 to 10.113
	DBP	7.816 ± 1.44	29.722	<0.001	7.278 to 8.354
	MAP	8.422 ± 1.105	41.757	<0.001	8.009 to 8.834

**Table 3:** Pearson's correlation coefficients between pairs of anthropometric characteristics in normotensive and hypertensive subjects.

Variable	Height	Weight	BMI	AC
Age (years)	0.535 **0.113**	0.784∗ **0.598**	0.701∗ **0.509**	0.668∗ **0.585**
Height (cms)		0.543 **0.165**	0.237 **0.305**	0.619∗ **0.021**
Weight (Kg)			0.934∗ **0.885**∗	0.743∗ **0.767**∗
BMI (Kg/m^2^)				0.617∗ **0.691**∗

*It indicates *P* < 0.001; bold values indicate correlations in anthropometric characteristics of hypertensive subjects.

**Table 4:** Results of Bartlett's test of sphericity and KMO.

Test		Normotensive subjects	Hypertensive subjects
Bartlett's test of sphericity	Approx. *χ* ^2^	231.012	119.48
	DF	10	10
	*P*	<0.0001	<0.0001

KMO measure of sampling adequacy		0.63	0.75

DF: degree of freedom.

**Table 5:** Eigenvalues and % of variation explained by each PC in normotensive and hypertensive subjects.

PCs	Normotensive subjects			Hypertensive subjects		
	Eigenvalue	Individual%	Cumulative%	Eigenvalue	Individual%	Cumulative%
1	3.59	71.84	71.84	3.0550	61.10	61.10
2	0.83	16.58	88.42	1.1249	22.5	83.60
3	0.32	6.34	94.76	0.4393	8.78	92.38
4	0.25	5.04	99.8	0.2830	5.66	98.04
5	0.01	0.2	100.00	0.0978	1.96	100

**Table 6:** Loadings of anthropometric characteristics in normotensive and hypertensive subjects.

Anthropometric characteristics	Loadings after varimax rotation							
	Normotensive subjects				Hypertensive subjects			
	PC1	PC2	PC3	PC4	PC1	PC2	PC3	PC4
Age	0.0004	−0.0006	−0.0000	**−1.0000**	−0.0036	0.0020	**0.9988**	−0.0026
Height	−0.0139	**−0.9676**	0.0002	−0.0008	0.0058	**0.9968**	0.0020	0.0043
Weight	**−0.6569**	−0.1812	−0.0008	0.0039	**0.6576**	0.0561	−0.0349	−0.0754
BMI	**−0.7538**	0.1757	0.0008	−0.0039	**0.7533**	−0.0566	0.0352	0.0760
AC	−0.0001	0.0001	**−1.0000**	−0.0000	0.0078	−0.0043	0.0027	**−0.9942**

**Table 7:** Pearson's correlation coefficient among all pairs of PCs in normotensive and hypertensive subjects.

PC	PC2	PC3	PC4
PC1	−0.00000225 **0.00000878**	0.0000000798 **0.00000423**	−0.0000167 **0.00000659**
PC2		−7.237*e* − 016 **0.00000919**	5.808*e* − 016 **0.0000142**
PC3			−7.557*e* − 017 **0.0000175**

Bold values indicate correlations in anthropometric characteristics of hypertensive subjects.

**Table 8:** Optimal values of *γ* and *σ* ^2^.

Parameters	Normotensive subjects		Hypertensive subjects	
	SBP	DBP	SBP	DBP
*γ*	369.9717	1.7430*e* + 004	1.7498*e* + 008	3.0844*e* + 006
*σ* ^2^	0.5882	7.0498*e* − 006	4.7587*e* − 004	2.3885*e* − 006

**Table 9:** Statistical indices for different models.

Model	Normotensive subjects						Hypertensive subjects					
	SBP			DBP			SBP			DBP		
	*R* ^2^ (%)	RMSE	MAPE	*R* ^2^ (%)	RMSE	MAPE	*R* ^2^ (%)	RMSE	MAPE	*R* ^2^ (%)	RMSE	MAPE
PCA-ANN	67.44	0.58	37.09	62.39	0.62	40.77	59.50	0.68	9.2	53.19	0.86	11.02
PCA-ANFIS	80.04	0.56	9.82	79.37	0.52	12.27	87.02	0.41	2.97	86.11	0.4	2.71
PCA-LS-SVM	95.42	0.21	5.88	94.22	0.24	4.05	98.76	0.11	0.88	98.78	0.11	0.84

**Table 10:** Comparison of results with other studies.

Ref.	Model developed	Predicted parameter	Remarks
[33]	FIR and ANFIS	Mean BP and AEP during anaesthesia	No significant difference between the results of two models
[34]	ANN and multiple linear regression (MLR)	SBP	ANN outperformed MLR
[35]	RS-SVM	BP	Training rapidity and accuracy of the RS-SVM model are both evidently improved
[36]	PCA-ANFIS, conventional maximum amplitude algorithm	SBP and DBP	PCA-ANFIS outperformed
[37]	FIS	Effect of aerobic exercise on BP	Preliminary validation results of the performance of the FIS are promising
Our study	PCA-LS-SVM, PCA-ANN, and PCA-ANFIS	SBP, DBP, and MAP	PCA-LS-SVM outperformed PCA-ANN and PCA-ANFIS
